# Synthesis of Free-Standing Flexible rGO/MWCNT Films for Symmetric Supercapacitor Application

**DOI:** 10.1186/s11671-019-3100-1

**Published:** 2019-08-06

**Authors:** Amit Kumar, Nagesh Kumar, Yogesh Sharma, Jihperng Leu, Tseung Yuen Tseng

**Affiliations:** 1 Department of Materials Science and Engineering, National Chiao Tung University, Hsinchu, 300 Taiwan; 2 Institute of Electronics, National Chiao Tung University, Hsinchu, 300 Taiwan; 3 Centre of Nanotechnology, I.I.T. Roorkee, Roorkee, 247667 India

**Keywords:** Graphene, Reduced graphene oxide, MWCNTs, Specific capacitance, Supercapacitor, Energy and power densities

## Abstract

Herein, we report a novel, simple, and cost-effective way to synthesize flexible and conductive rGO and rGO/MWCNT freestanding films. The effects of MWCNT addition on the electrochemical performance of rGO/MWCNT nanocomposite films are investigated in some strong base aqueous electrolytes, such as KOH, LiOH, and NaOH via three-electrode system. The supercapacitor behavior of the films is probed via cyclic voltammetry, galvanostatic charging-discharging, and electrochemical impedance spectroscopy. The structural and morphological studies of the films are performed by X-ray diffractometer, Raman spectrometer, surface area analyzer, thermogravimetric analysis, field emission scanning electron microscope and transmission electron microscope. The rGO/MWCNT film synthesized with 10 wt% MWCNTs (GP10C) exhibits high specific capacitance of 200 Fg^−1^, excellent cyclic stability with 92% retention after 15,000 long cycle test, small relaxation time constant (~ 194 ms), and high diffusion coefficient (7.8457 × 10^−9^ cm^2^ s^−1^) in 2 M KOH electrolyte. Furthermore, the symmetric supercapacitor coin cell with GP10C as both anode and cathode using 2 M KOH as electrolyte demonstrates high energy density of 29.4 Whkg^−1^ and power density of 439 Wkg^−1^ at current density 0.1 Ag^−1^ and good cyclic stability with 85% retention of the initial capacitance at 0.3 Ag^−1^ after 10,000 cycles. Such a high performance of the GP10C film in the supercapacitor can be ascribed to the large surface area and small hydration sphere radius and high ionic conductivity of K^+^ cations in KOH electrolyte.

## Introduction

Graphene, due to its extraordinary physical properties such as very high specific surface area, exceptional electrical conductivity, excellent mechanical flexibility, and unusual thermal/chemical stability, has become one of the most studied materials in material science after its discovery in 2004 [1–3]. Because of aforementioned unique properties, graphene has found potential applications in nanoelectronics [4], sensing [5], energy storage [6], solar cells [7], and nanomechanical devices [8]. However, the fabrication of uniform large area film of monolayer or bilayer pristine graphene is not only difficult but expensive too, which hinders its commercialized applications in device fabrication. Therefore, researchers use reduced graphene oxide (rGO), derived by the chemical and/or thermal reduction of hydrophilic graphene oxide (GO), as an alternate to pristine graphene. Recently, the demand for cheap, reliable, portable, and bendable electronic devices has increased tremendously [9]. In this regard, flexible energy storage devices (supercapacitors and Li-ion batteries) have become the center of attraction for the world scientific community because of their aim for integration within flexible electronic devices [10–15]. In this regard, materials which can be easily transformed into free-standing paper-like form are highly desirable. Therefore, when searching for such a bendable material that possesses good mechanical and chemical stability, excellent electrical conductivity and easy to transform into large-area thin film, rGO is found to be a highly promising and propitious candidate [16, 17]. There were two approaches to prepare free-standing rGO paper-like film or membrane. The first approach involves the direct filtration of rGO dispersion over specific filter papers [18, 19]. The second approach starts with the synthesis of GO powder and complete with the reduction of GO paper into rGO paper either using some reducing agent or via annealing in inert/reducing environment [20–23]. Various techniques have been reported to synthesize free-standing flexible rGO paper. Xiao et al. fabricated rGO paper by printing technique followed by CO_2_ bubbling delamination method and the obtained paper showed the specific capacitance of 55 Fg^−1^ at 1 Ag^−1^ [20]. Rath et al. synthesized rGO paper via vacuum filtration of GO suspension and subsequent reduction using hydriodic acid (HI) (55%) and obtained the specific capacitance (SC) of ~ 80 Fg^−1^ at 0.5 Ag^−1^ [21]. Li et al. documented the SC of 130 Fg^−1^ at 0.1 Ag^−1^ for the rGO paper prepared by vacuum filtration of GO aqueous suspension followed by reduction via Zn powder in ammonia solution [22]. Further, Hu et al. synthesized rGO paper by vacuum filtration of GO aqueous dispersion and subsequent electrochemical reduction. They reported the SC of 106 F cm^−3^ at 1 mV s^−1^ scan rate [23]. Based on literature evidences, it has been concluded that *π*-*π* interaction and strong van der Waals interactions between basal planes cause restacking and aggregation of the rGO nanosheets, which result in reduced surface area and poor electrochemical performance of the rGO paper [24–27].

In this study, we report a novel, facile, and cost-effective way to synthesize flexible conducting rGO film with multiwall carbon nanotubes (MWCNTs) intercalated between the rGO sheets. We indicate that use of an appropriate amount of MWCNTs to form rGO/MWCNT nanocomposite film can effectively prevent the restacking of rGO nanosheets, therefore, improve the electrochemical performance of the films. An optimum amount of HI, followed by annealing at 250 °C in reducing environment (3% H_2_ + 97% N_2_) for 2 h, is used for the reduction of GO/MWCNTs to rGO/MWCNT films. The thickness of the films can be controlled easily just by tuning the volume of GO dispersion used in the synthesis of rGO and rGO/MWCNT films. We examine the electrochemical performance of rGO/MWCNT nanocomposite flexible films fabricated with various wt% (0, 5, 10, and 15) MWCNTs. Results show that the rGO/MWCNT film synthesized with 10 wt% MWCNTs exhibits excellent specific capacitance of 200 Fg^−1^ at 0.25 Ag^−1^ in 2 M KOH aqueous electrolyte, higher than several previous reported values. As-prepared optimized free-standing nanocomposite films were used as both anode and cathode for designing a symmetrical supercapacitor device that exhibits high energy density of 29.4 Whkg^−1^ and good stability with 85% retention after 10,000 cycles in 2 M KOH aqueous electrolyte.

## Methods

### Materials

All the chemicals used in this study were analytical pure grade. Natural graphite fine powder (No. 15553, Riedel-de Haen), MWCNTs (Ctube-120, length 5–20 μm) were received (CNT Co., Ltd., South Korea). Hydriodic acid (57% w/w aq. soln.) was purchased from Alfa Aesar. Polyvinyl alcohol (PVA, MW 89,000~98,000) was purchased from Sigma-Aldrich Company. All dispersions and solutions were prepared in DI water of resistivity at least 18 MΩ cm at 25 °C, obtained from Milli-Q water purification system (Milli-Q, USA).

### Preparation of Graphene Oxide

The precursor material, graphene oxide (GO) was synthesized by the strong chemical oxidation of graphite powders in a mixture (9:1) of H_2_SO_4_ and H_3_PO_4_ [28]. The obtained product (GO flakes) was vacuum dried at 45 °C to remove moisture.

### Purification of MWCNTs

Before utilization, the commercially available MWCNTs (specific surface area, 40–300 m^2^ g^−1^; length, 5–20 mm) were refluxed in 70% nitric acid solution at 90 °C for 24 h. After reflux, the resultant mixture was filtered over nylon membrane filter (0.45 mm) and washed with excess amount of DI water until pH turns neutral. The filtered solid was dried in an oven at 100 °C for 24 h to obtain purified and functionalized MWCNTs.

### Synthesis and Fabrication of rGO/MWCNT Flexible Freestanding Films

**Fig. 1:**
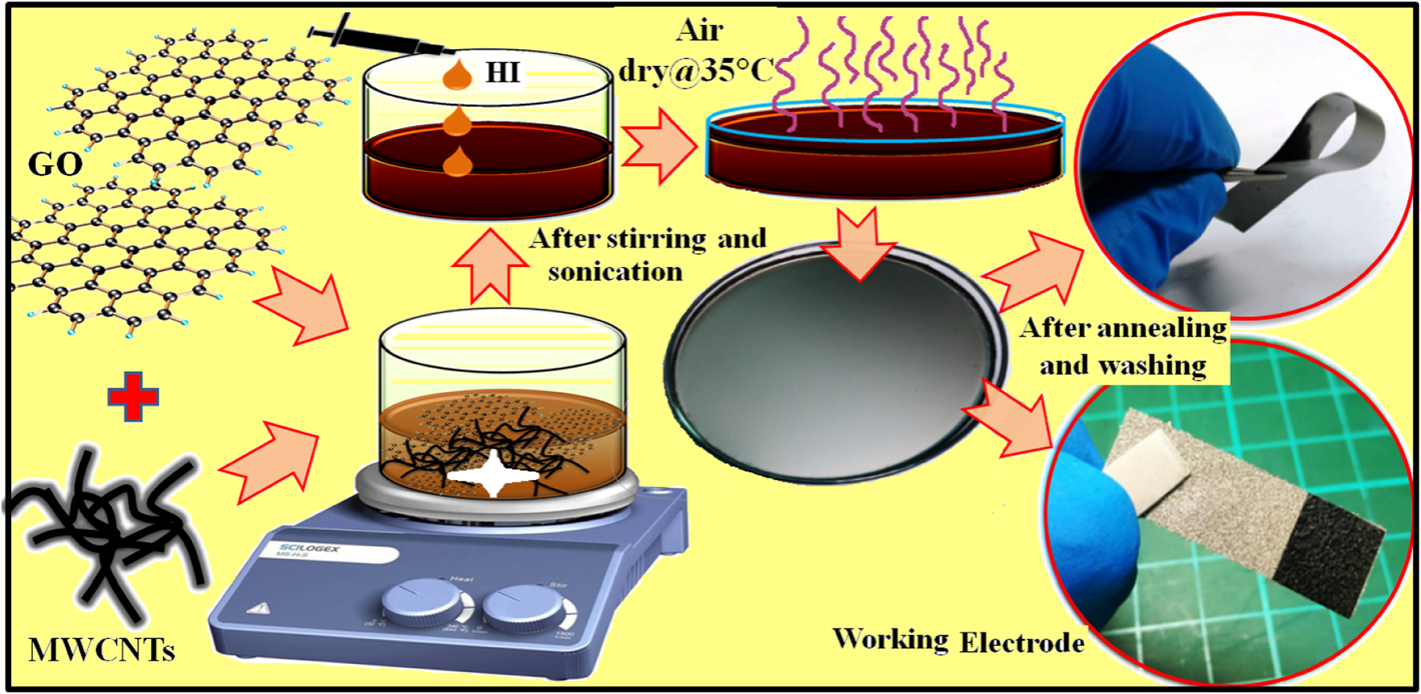
Scheme of the synthesis of rGO, rGO/CNT papers, and electrode fabrication process

To synthesize rGO/MWCNT films, calculated amount of GO flakes were dispersed well in DI water via intense sonication to prepare homogeneous GO dispersion of 8 mg/mL. After that, 0, 5, 10, and 15 wt% of MWCNTs were mixed with optimized quantity (20 mL) of GO dispersion separately via ~ 1 h intense sonication. An optimum amount of HI solution, as a reducing agent, was added into the above GO-MWCNT mixture drop by drop. The resultant mixture was poured into a petri dish of diameter ~ 9.5 cm and dried in the airflow. The dried rGO/MWCNT film can easily be removed from the petri dish in the presence of ethanol. Thus, the obtained free-standing rGO/MWCNT film was washed several times with ethanol to remove unreacted/residual HI solution and again dried in air at 35 °C for 12 h. Finally, the air-dried free-standing film was annealed at 250 °C in reducing environment (3% H_2_ + 97% N_2_) for 2 h. The schematic of whole synthesis process is illustrated in Fig. 1. The fabricated rGO/MWCNT films with different MWCNT amounts, 0, 5, 10 and 15 wt%, are termed as GP, GP5C, GP10C, and GP15C, respectively.

### Fabrication of rGO/MWCNT Electrodes

The working electrodes of rGO/MWCNT films for electrochemical testing were prepared by pressing a piece (1 × 1cm^2^) of fabricated film onto Ni foam with a uniform ~ 10 mPa pressure for 2 min. The weight of active material loaded on Ni foam substrate, as measured by the microbalance (PRECISA XR125M-FR) with an accuracy of ~ 0.1 μg, was ~ 1.1 mg. Synthesis process and electrodes fabrication are shown in Fig. 1.

### Fabrication of GP10C Film-Based Symmetric Coin Cell and Solid-State Flexible Devices

The GP10C electrode-based symmetric supercapacitor was successfully designed in a two-electrode coin cell configuration using 2 M KOH electrolyte. Briefly, two circular GP10C electrodes of equal weights were punched into CR2032 coin cell assembly. Here, in order to prevent direct contact of the working electrodes, a separator (Glass microfiber membrane, Whatman^TM^) was sandwiched between them. The total mass of active material in the device was ~ 3.5 mg. Further to see the compatibility of GP10C electrode material in a flexible device, a flexible solid-state symmetric device (FSSSD) was designed using PVA-KOH gel polymer electrolyte. For the preparation of FSSSD, 1 g PVA was dissolved in 5 mL DI water at 85 °C and stir for 1 h until the solution becomes transparent, after that, 1 g of 2 M KOH solution was added into the above solution. Finally, the mixture was left for 3 h with continuous stir to get quasi-solid gel-like form [29]. For device assembling, two pieces (1 × 2cm^2^) of GP10C electrodes of equal weights were attached onto flexible stainless steel fabrics, which prevent the electrodes from mechanical shock and provide them support for external contact. Both working electrodes were uniformly coated with quasi-solid like gel electrolyte. To get a proper solid gel-like layer, both working electrodes were air-dried in fume hood to remove access of water, sandwiched together face to face, and finally wrapped by adhesive tape.

### Physico-Chemical Characterizations and Electrochemical Measurements

The prepared rGO/MWCNT films were carefully examined via X-ray diffractometer (XRD, BRUKER D2 PHASER) assembled with CuKα irradiation (*λ* = 1.54184 Å, 10 mA and 30 kV), and field emission scanning electron microscope (FE-SEM, Hitachi SU8010) for performing crystalline and surface morphology analysis, respectively. Raman spectra measurement of the samples was carried out using 514.5 nm Ar laser, 40 mW (Horiba Jobin Yvon Labarm HR 800). Brunauer-Emmett-Teller (BET) surface area analyzer (BET, ASAP 2020) was used to identify specific surface area. Thermal gravimetric analysis (TGA) was performed from 30 to 900 °C at 3 °C min^−1^ ramping rate under N_2_ environment using thermogravimetric analyzer (TGA, TA Instruments Q500). The Ohmic resistances of the as-synthesized films were measured via four-point probe method (NAPSON RT-7), and the electrical conductivity is calculated using the following equation:

1 $$ \sigma =\frac{l}{\mathrm{Rs}\times A} $$

where *σ, l, A,* and Rs, respectively, represents the electrical conductivity, thickness, cross-sectional area, and Ohmic resistance of the synthesized film as measured via four-point probe instrument. The electrochemical properties of rGO/MWCNT film electrodes were investigated by cyclic voltammetry (CV), galvanostatic charge/discharge (GCD), and electrochemical impedance spectroscopy (EIS) using CHI instrument 616B electrochemical analyzer at room temperature. A three-electrode configuration, which contains saturated calomel reference electrode (SCE), platinum sheet as a counter electrode, and rGO/MWCNT film as working electrode were utilized for these measurements in electrolytes of KOH, LiOH, and NaOH. The SC (Cs) from GCD curve is calculated using the following equation:

2 $$ C=\frac{I\ \Delta  t}{m\ \Delta  V} $$

where *I* is the discharge current, *∆t* is the time for a full discharge, *m* is the mass of active electrode material, and *∆V* represent*s* the width of a potential window for a full discharge.

The electrochemical impedance spectroscopy (EIS) results were obtained by applying an ac amplitude of 5 mV in the frequency range from 0.1 Hz to 100 KHz and measuring the amplitude and the phase shift of the resulting current. Preferably, a supercapacitor can be symbolized by a simple circuit having a resistor in series with a capacitor. Here, resistor and capacitor represent the equivalent series resistance (ESR) and the capacitance of the device, respectively. The net impedance of this circuit can be expressed as;

3 $$ {Z}_{\mathrm{RC}}=R+1/ j\omega C $$

where, ω = 2*πf* and *f* = frequency in Hz. Equation (3) shows that at higher frequency values ESR term is dominant, while at lower frequency values capacitive term becomes more effective, and the system starts behaving like a pure capacitor. Further, EIS data analysis provides frequency-dependent characteristic of the supercapacitor electrode materials in terms of complex power as given below:

4 $$ S\left(\omega \right)=\mathrm{P}\ \left(\upomega \right)+\mathrm{iQ}\ \left(\upomega \right) $$

where *P* (*ω*), real component of power, is defined as active power (watt), and *Q* (*ω*), i.e., an imaginary component of power, is termed as reactive power (volt-ampere-reactive, VAR).

*P* (*ω*) and *Q* (*ω*) can be written as follows:

5 $$ P\ \left(\omega \right)=\left[\ \Delta  {V^2}_{\mathrm{rms}}/|Z\ \left(\omega \right)|\right].\cos\ \upphi $$

6 $$ Q\ \left(\omega \right)=\left[\ \Delta  {V^2}_{\mathrm{rms}}/|Z\ \left(\omega \right)|\right].\sin\ \upphi $$

The above equations (4)–(6) can be directly used to find out the power values of the supercapacitor.

Further, the diffusion coefficients of the electrolytic ions in the film electrode at the interfacial region have been determined using Randles plots, which are the linear plots of the real part (*Z′*) and or imaginary part (*Z″*) of the impedance (*Z*) versus *ω*^−1/2^. The slope of the Randles plot is used to find out the Warburg coefficient (*σ*), which is related to the following given below [30];

7 $$ \sigma =\frac{RT}{n^2{F}^2A\sqrt{2}}\left(\frac{1}{C^{\ast}\sqrt{D}}\right) $$

where *T* is the absolute temperature, *n* is the charge-transfer number, *R* represents the gas constant, C* is the concentration of the electrolyte, and *A* represents the area of working electrode.

## Results and Discussion

**Fig. 2:**
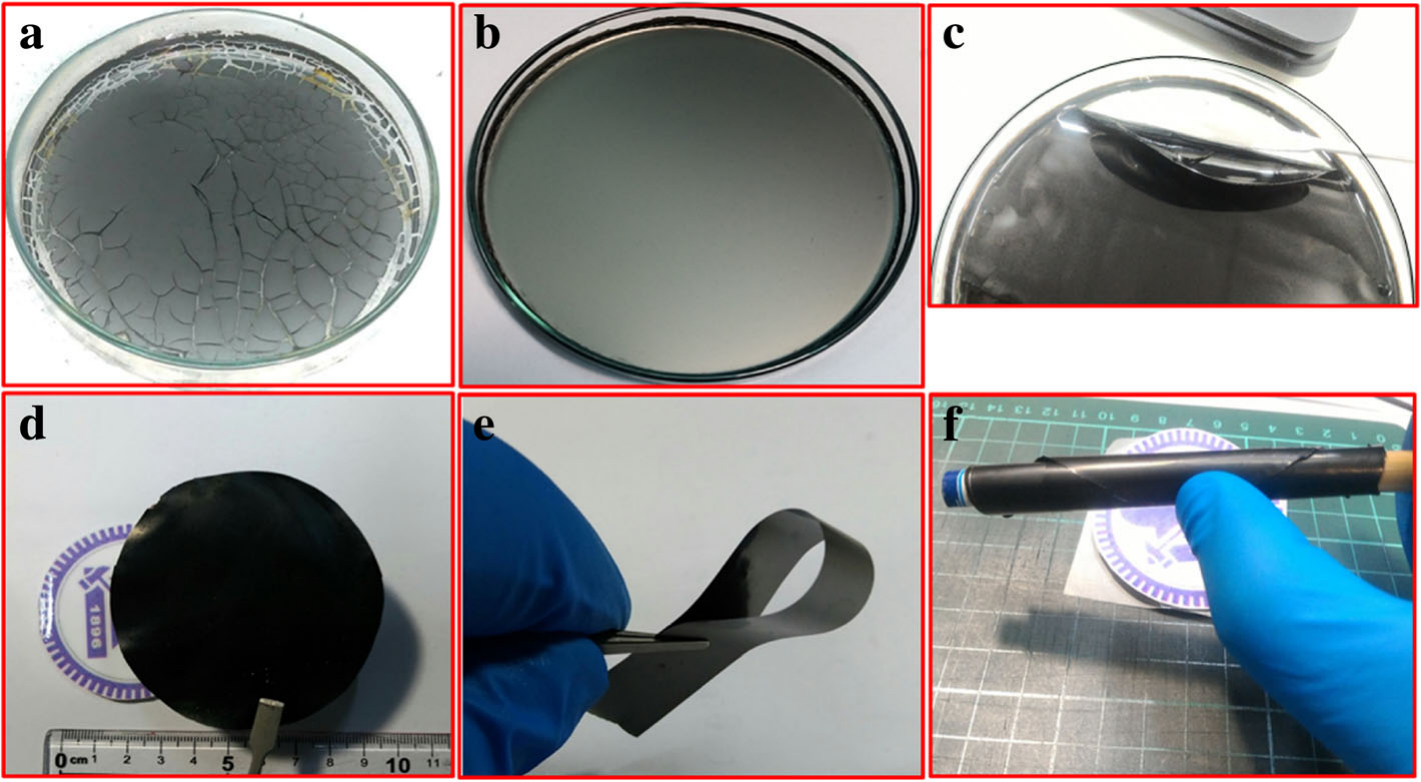
**a** Cracked rGO/MWCNT film, **b** uniform film, **c** uniform film removed from the petri dish, and **d**–**f** washed and annealed free-standing film

We synthesized the rGO/MWCNT-based nanocomposite films via efficient one-step chemical route. Generally, rGO-based nanocomposites are well known for energy storage materials. Moreover, as reported in the literature, the MWCNTs were utilized to establish a conductive channel inside the material [31]. Therefore, we study the effect of incorporation of MWCNTs on the electrochemical performance of the free-standing rGO-based films. We observe that the amount of HI (reducing agent) is crucial in order to obtain continuous conducting free-standing rGO/MWCNT films. A little more quantity than the optimum value will leave cracks in the film as excess amount of HI causes more I_2_ to liberate (HI + H_2_O → H_3_O^+^ + I^−^, and 2I^−^ = I_2_ + 2e^−^), which would cause cracks in the film as shown in Fig. 2.

### Structural and Morphological Characterizations

**Fig. 3:**
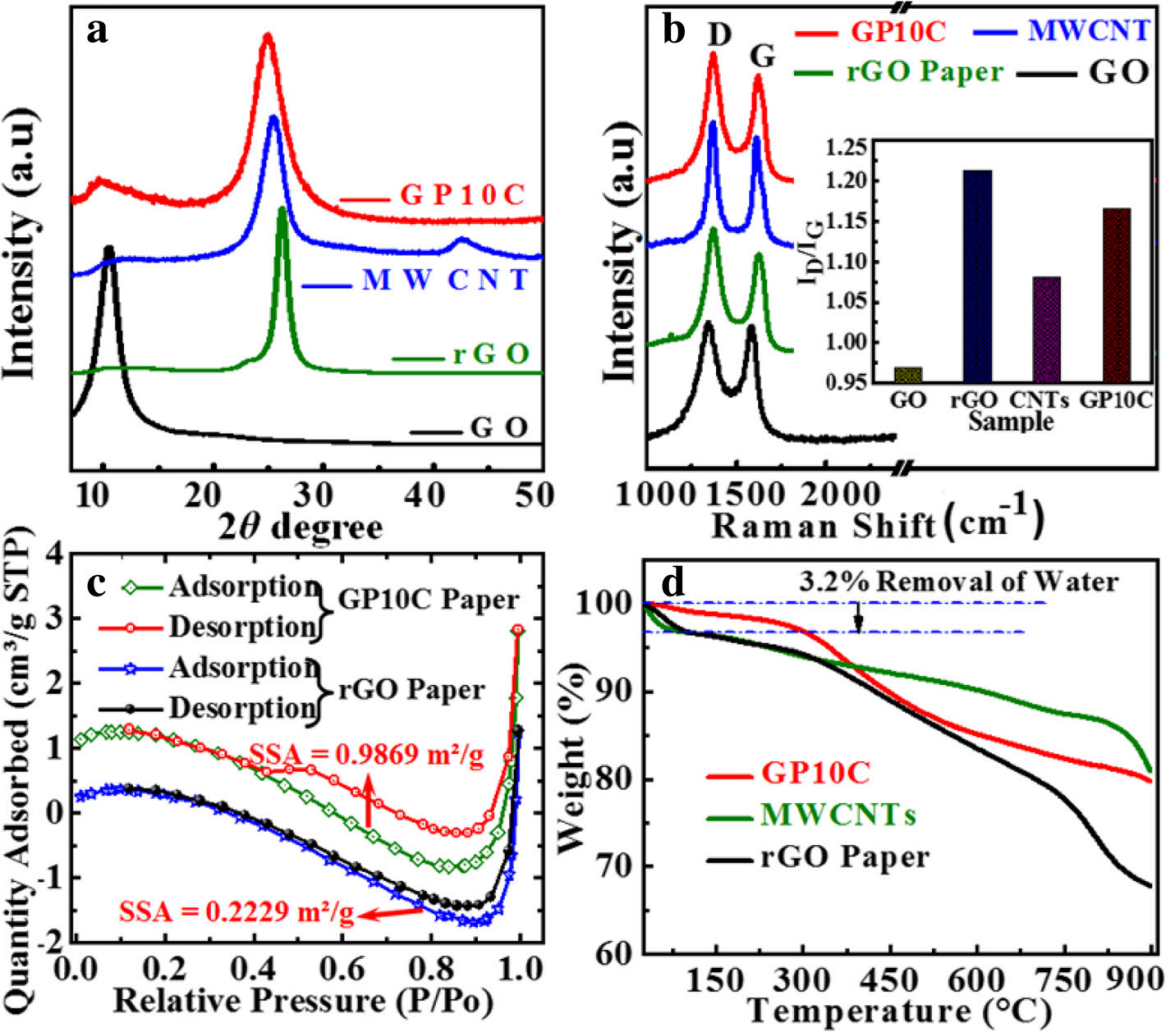
XRD patterns of GO, rGO paper, MWCNTs, and GP10C film. **a**, **b** Raman spectra evolution of D and G bands, **c** BET analyses of rGO, rGO/CNT films, and **d** TGA curves of rGO film, MWCNT, and GP10C film

XRD patterns of the GO, rGO films, MWCNT, and GP10C are shown in Fig. 3 a. The comprehensive characterization of XRD represents the deoxygenation of the as-prepared films. The XRD pattern of GO film indicates that a sharp diffraction peak at 2*θ* = 10.4°, corresponds to the characteristic (001) diffraction of GO. This suggests larger interlayer spacing (*d* = 0.8465 nm) of GO than that of graphite (~ 0.34 nm) due to the introduction of oxygen-containing functional groups (e.g., epoxy and hydroxyl groups) adhered on the GO sheet surface and to the presence of a single-molecule-thick layer of water molecules intercalated between the sheets [32–34]. In case of rGO, MWCNTs, and GP10C samples, the diffraction peaks appear at 2*θ* = 26.24°, 25.49°, and 25°, respectively. The successful reduction of graphene oxide is evident by the significant shrinkage of interlayer spacing in rGO (~ 0.3475 nm) and GP10C (~ 0.36 nm), attributed to the destruction of oxygen-containing functional groups. The Raman analyses of rGO/MWCNT films (Fig. 3b) are executed to further explore the structures of GO, rGO, MWCNTs, and GP10C by the resulting characteristic G and D bands related to defects and disorder, respectively. To observe the defects presented in graphene-related materials, the intensity ratio (*I*_D_/*I*_G_) for the D band (at 1350 cm^−1^) and the G band (at 1590 cm^−1^) is generally used [35]. The I_D_/I_G_ ratio (inset, Fig. 3b) increases from 0.9685 for GO film to 1.2123, 1.0807, and 1.1649 for rGO paper, MWCNTs, and GP10C, respectively, indicating more defects in rGO, MWCNT, and GP10C films than in pure GO film. Enhancement in the defects is probably due to the disintegration of graphene sheets into smaller sp^2^ graphene domain and the loss of carbon atoms induced by the decomposition of oxygen-containing groups [36]. The value of *I*_D_/*I*_G_ ratio for GP10C film is smaller (1.1649) than that of rGO film (1.2123) that can be ascribed to the increment in sp^2^ domains caused by introducing carbon nanotubes [37]. The N_2_ adsorption-desorption isotherms of rGO and GP10C films after applying uniform pressure of 10.0 MPa for 5 min are shown in Fig. 3 c. The calculated BET specific surface area for GP10C (0.9869 m^2^/g) is found more than 4 times higher than that of rGO film (0.2229 m^2^/g). The higher specific surface area predicts the availability of more interfacial area between the electrolytic ions and the electrode active material and might provide better electrochemical performance [38]. The higher specific surface area can be attributed to the MWCNTs sandwiched between rGO layers, which prevent the restacking of rGO sheets upon applying external pressure. In order to investigate the thermal stability, TGA of the synthesized films is accomplished in N_2_ environment at the ramping rate of 3 °C min^−1^ from 30 to 900 °C (Fig. 3d). In the TGA graphs, 3.2% weight loss from 30 to 255 °C is related to the evaporation of surface absorbed water and to the removal of interlayered water molecules [39]. The weight loss of about 18.6% in the range of 302 to 810 °C can be attributed to the decomposition of hydrophilic functional groups, attached with the rGO and MWCNTs during purification and synthesis processes and related to the thermal decomposition of reduced graphene oxide and carbon nanotubes [40]. We observe that thermal stability of GP10C film is better than that of pure rGO film, which can purely be ascribed to the presence of MWCNTs in the free-standing GP10C.

**Fig. 4:**
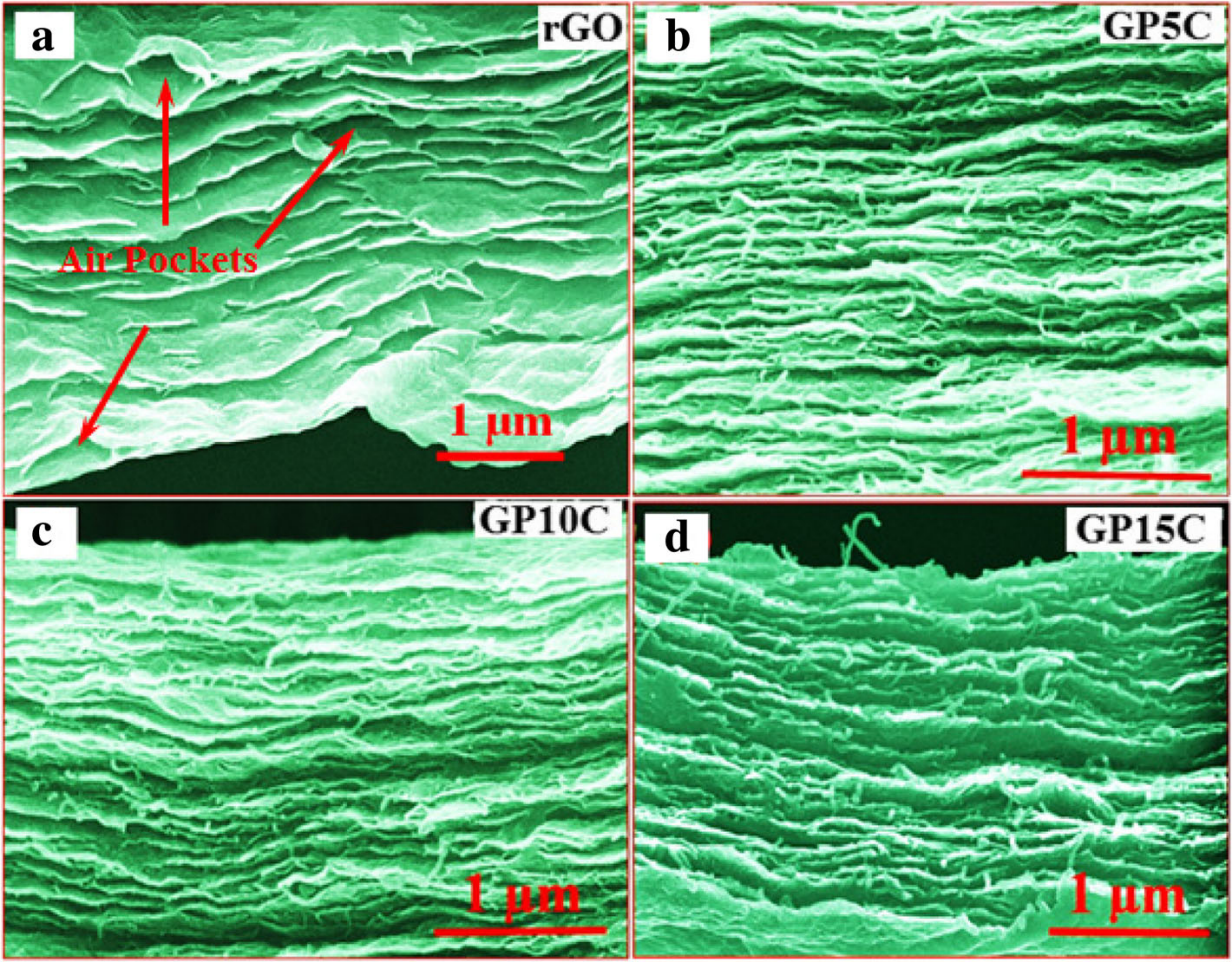
Cross-sectional FE-SEM images of **a** rGO film, with different MWCNTs loading **b** 5 wt.%, **c** 10 wt.%, and **d** 15 wt.%

The FESEM micrographs of the rGO and rGO/MWCNT films are shown in Fig. 4. The cross-sectional examination (Fig. 4a) reveals that rGO sheets are aligned and restacked on top of the other in the rGO film. We observe the presence of some air pockets between the rGO layers, which arises due to the liberation of oxygen and other gaseous species during reduction and annealing process. These air pockets diminish the electrical conductivity and hence the electrochemical performance of the free-standing film [41]. We observe with the addition of MWCNTs in the film (Figs. 4b–d), rGO layers become more aligned with lesser air pockets as MWCNTs working as a filler and providing an alternating path for gas species to come out from the film.

### Electrical Conductivity Measurements

**Fig. 5:**
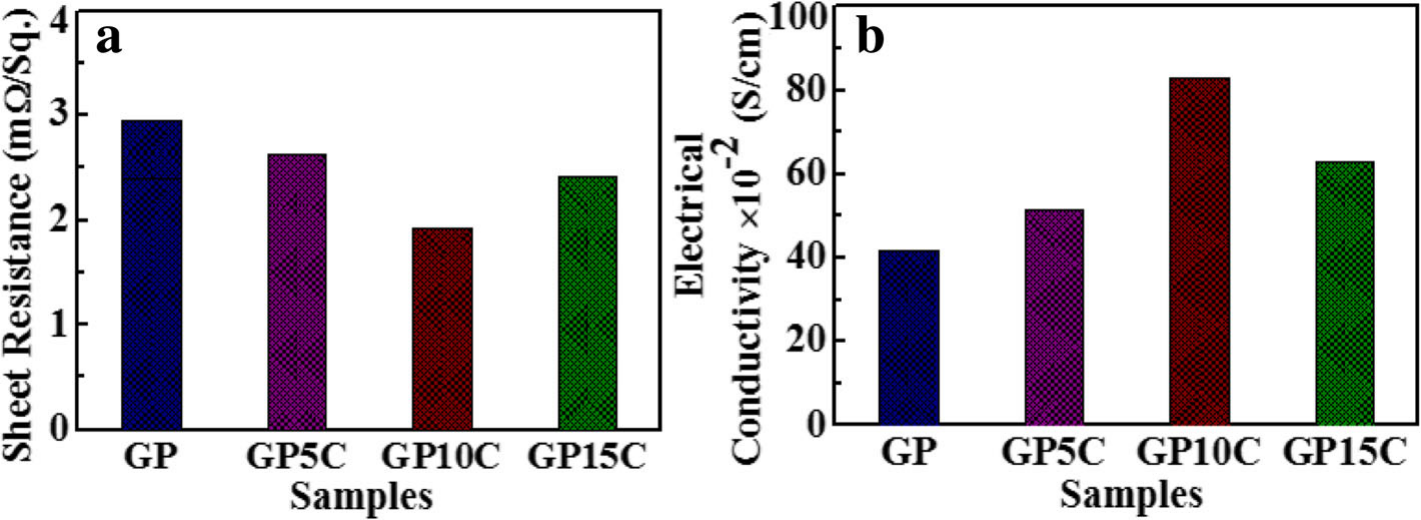
**a** Ohmic resistances of rGO and rGO/MWCNT papers with 5, 10, and 15 wt.% MWCNT content, and **b** the electrical conductivities of the same

The electrical conductivity is a very important parameter to investigate the electrochemical performance of the as-prepared rGO and rGO/MWCNT films. The electrical measurements of GP, GP5C, GP10C, and GP15C with thicknesses about 0.01, 0.015, 0.014, and 0.0165 mm, respectively, were conducted via four-point probe instrument and the measured Ohmic resistances of GP, GP5C, GP10C, and GP15C are found to be 2.94, 2.71, 1.93, and 2.66 mΩ/sq., respectively (Fig. 5a). Figure 5b depicts the values of electrical conductivity calculated by Eq. (1) for GP, GP5C, GP10C, and GP15C to be 41.7 × 10^−2^, 51.4 × 10^−2^, 82.9 × 10^−2^, and 62.9 × 10^−2^ S cm^−1^, respectively. The electrical conductivity of the films increases with an increase of MWCNT ratio from 0 to 10 wt.%. This can be attributed to the presence of electrical conducting network formed by MWCNTs in the films. The addition of MWCNTs in the rGO film allows the formation of a 3D network, which works as a conducting channel for charge-transportation inside the film and hence improves its electrical conductivity. As the loading of MWCNTs in the rGO increases, the alignment of MWCNTs becomes less pronounced (Fig. 4b–d). At higher MWCNT concentration (15 wt.%), the agglomerating tendency of MWCNTs between rGO layers becomes effective that reduces the conductive network formation of MWCNTs throughout the film, and hence the value of electrical conductivity decreases [42]. This is basically caused by the effect of increasing contact resistance [43, 44]. Among various synthesized films, GP10C exhibits a lower value of Ohmic resistance (1.93 mΩ/sq.) with higher electrical conductivity of 82.9 × 10^−2^ S cm^−1^. The enhancement in the electrical conductivity of GP10C is a result of the strong *π*-*π* coupling between rGO and MWCNTs that boosts more mobile charge careers delocalization between the electronic densities of both [45].

### Electrochemical Performances of GP10C Film in Various Alkaline Electrolytes

**Fig. 6:**
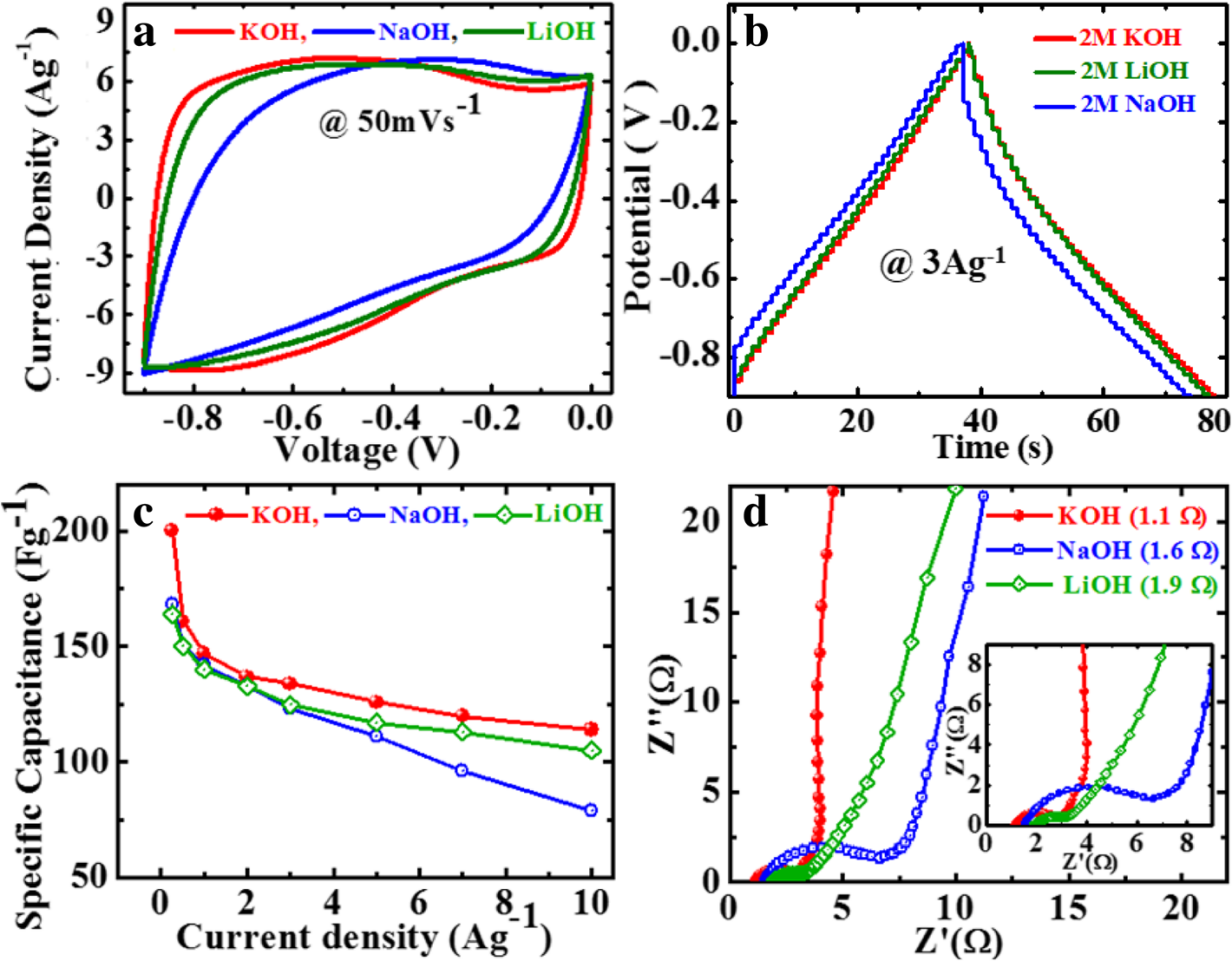
Electrochemical performance of GP10C film in different aqueous KOH, LiOH, and NaOH in 2 M electrolytes, **a** CV curves at 50 mVs^−1^, **b** GCD curves at 3 Ag^−1^, **c** SC as calculated from GCD curves, and **d** Nyquist plot in various electrolytes with inset showing the magnified region

The electrochemical properties measurements of the GP10C films were carried out in aqueous electrolytes via CV, GCD, and EIS at room temperature. Electrolyte is one of the most important factors that greatly influence the electrochemical properties of a supercapacitor. Therefore, to find the best suitable alkaline electrolyte for the film electrodes, we investigate the electrochemical performance of GP10C electrode in three most commonly used alkaline *electrolytes*, namely, KOH, NaOH, and LiOH, and the results are shown in Fig. 6. For different electrolytes, CV curves occupy different areas (Fig. 6a). Noticeably, the CV curve of GP10C is nearly rectangular in shape and occupies a larger area in KOH than those in NaOH and LiOH when measured at the scan rate of 50 mVs^−1^. In Fig. 6b, the GCD curves of GP10C at the current density of 3 Ag^−1^ show longer discharge time in KOH as compared to those in NaOH and LiOH electrolytes. It is obvious from Eq. (2) that longer the discharge time (Δ*t*), the higher the SC would be. Therefore, we obtain higher SC in 2 M KOH as compared to those in 2 M LiOH and NaOH electrolytes (Fig. 6c). The observed asymmetry in the GCD curves (Fig. 6b) arises due to the occurrence of some faradic reactions at the surface of composite films. This phenomenon can be ascribed to the oxygen-containing functional groups attached with the rGO sheets and functionalized MWCNTs. The EIS is basically used to execute the electrochemical performance of the films in terms of ion transfer and electrical conductivity. The Nyquist plots of GP10C in different electrolytes are examined in the frequency range from 0.1 Hz to 100 KHz with ac amplitude of 5 mV (Fig. 6d). The Nyquist plot of GP10C contains basically two major components (real part Z*′* and imaginary part Z*″*) representing a complex plane in which Z′ exhibits the Ohmic behavior; on the other hand, Z″ shows the capacitive behavior of the film electrode. It can be explained theoretically via three frequency-dependent regions, namely, high-frequency region (impedance arc), low-frequency region, and middle-frequency region (Warburg impedance).

**Fig. 7:**
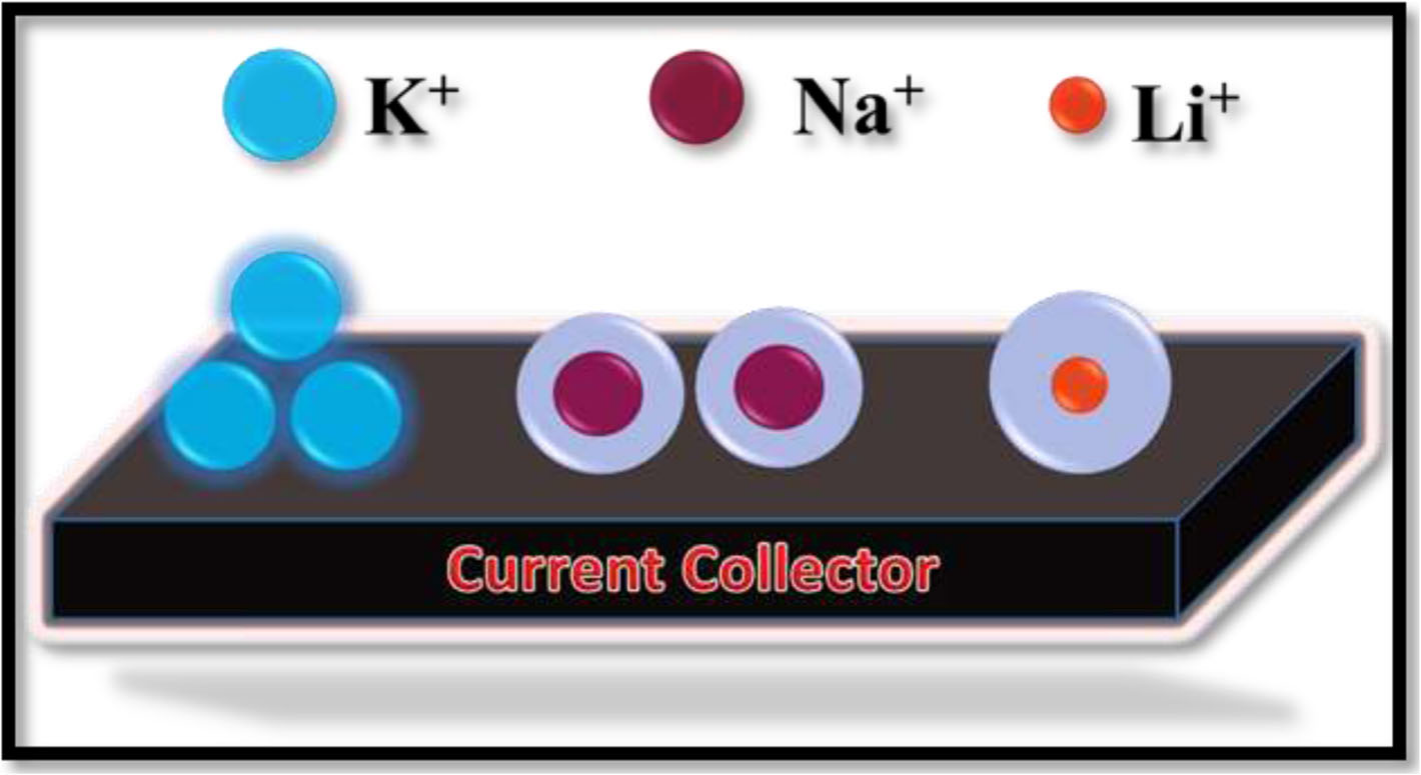
Schematic diagram of hydrated ionic radii of the ions associated with different electrolytes used for GP10C electrode measurement

Supercapacitor works similar to pure resistor at higher frequency range, while at lower frequencies an acute increment in the imaginary part and nearly vertical line observed, exhibiting the pure capacitive behavior. The middle-frequency region represents the interaction between electrolytic ions and the porous active sites of film electrodes. Furthermore, in EIS, the ionic resistance of electrolyte, the internal resistance of current collector and active material, and electrode-current collector interface contact resistance play a key role to find out the effective series resistance (ESR) or solution resistance (Rs). In the high-frequency region of the Nyquist curve, ESR can be observed by the point value where the curve intersects the real axis. The value of Rs is found to be smaller (~ 1.1 Ω) for KOH than those measured for NaOH (~ 1.6 Ω) and LiOH (~ 1.9 Ω). It is also noteworthy to mention that the diameter of the semicircular arc in the high-frequency region and the length of inclined line at an angle of 45° in the middle frequency region are the representative of diffusion resistance and Warburg resistance, respectively. In this regard, GP10C exhibits smaller diffusion resistance and Warburg resistances in KOH, when compared with those of LiOH and NaOH [46, 47]. The excellent performance of the GP10C electrode in KOH may be associated to a smaller hydrated ionic radius and higher ionic conductivity of K^+^ ion (64.3 Ohm^−1^ cm^2^ mol^−1^) as compared to that of Na^+^ (43.5 Ohm^−1^ cm^2^ mol^−1^) and Li^+^ (33.5 Ohm^−1^ cm^2^ mol^−1^) ions. On the other hand, the ionic mobility enhanced by a lower hydrated ionic radius of K^+^ ion gains access to the electrode surface, resulting in an improved electrochemical performance of the GP10C electrode [48, 49]. A straightforward explanation of the K^+^, Na^+^, and Li^+^ ions with hydrated ionic radii, 232, 276, and 340 pm, respectively, is shown in Fig. 7. Rather than outer factors, the real ionic radius is found inversely proportional to the Coulomb force in light of formula *F* = *KQ*_1_*Q*_2_/*r*^2^, where *F* is Coulomb force, *r* is the distance between two charges (*Q*_*1*_ and *Q*_*2*_), and *K* is the Coulomb’s constant. The ionic radius follows the order of rK+ (= 138 pm) > rNa^+^ (= 102 pm) > r Li^+^ (= 76 pm), so the Coulomb force follows the order of K^+^ < Na^+^ < Li^+^. The larger Coulomb force will be united with a larger number of water molecules, making hydrated ionic radius larger [50, 51], therefore, K^+^ ion has a lower hydrated ionic radius. On the basis of the above results and discussion, KOH aqueous electrolyte is found as a mostly suitable electrolyte among the three studied electrolytes for the rGO/MWCNT film electrode.

### Electrochemical Performance of rGO/MWCNT Films

**Fig. 8:**
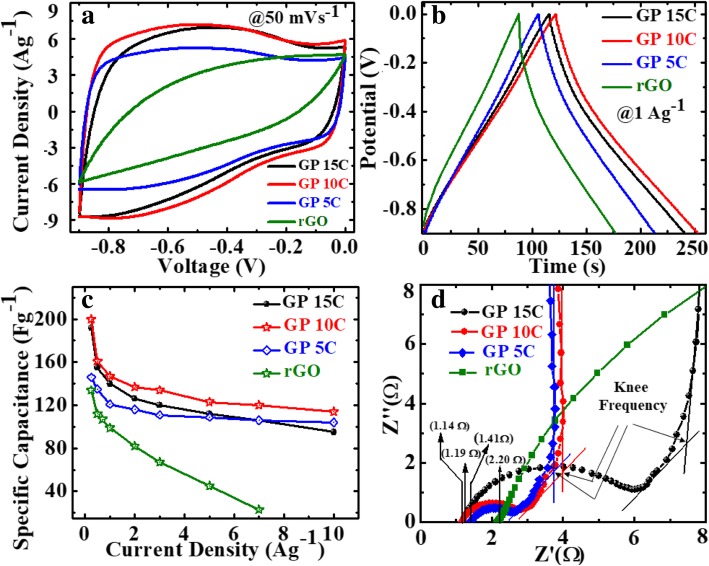
Electrochemical performance of rGO, GP5C, GP10C, and GP15C electrodes in 2 M KOH electrolyte, **a** CV curves at the scan rate of 50 mVs^−1^, **b** GCD curves at the current density 1 Ag^−1^, **c** CV as determined from GCD curves, and **d** Nyquist plots comparison of all the papers

We also investigated the effect of MWCNT addition on the electrochemical performance of rGO/MWCNT films in a three-electrode setup with 2 M KOH electrolyte. Figure 8a depicts the CV curves of as-synthesized rGO, GP5C, GP10C, and GP15C film electrodes recorded at a scan rate of 50 mVs^−1^ in the potential range − 0.9 to 0.0 V. Evidentially, in comparison to GP, GP5C, and GP15C, the CV curve of GP10C occupies the larger area, and it belongs to nearly rectangular shape, implying the electrical double-layer (EDL) capacitive behavior of this electrode with higher SC value [52]. Figure 8b represents the GCD curves of all the films recorded at 1 Ag^−1^ in the potential range − 0.9 to 0.0 V. Furthermore, similar to CV results, the charge/discharge curves being nearly triangular in shape also verify the electrical double-layer capacitor (EDLC) behavior of the film electrodes. Here, it is clear that the GP10C has significantly longer discharge time (∆*t*), and hence higher SC among the synthesized films. The values of CVs calculated from the GCD curves using Eq. (2) as function of discharge current densities are shown in Fig. 8c. The GP10C exhibits specific capacitances of 200, 161, 147, 137, 134, 123, 120, and 114 Fg^−1^ at 0.25, 0.5, 1, 2, 3, 5, 7, and 10 Ag^−1^, respectively, and it is able to maintain ~ 57% of its initial capacitance value (200 Fg^−1^) from 0.25 to 10 Ag^−1^. The specific capacitance of rGO increases significantly after the addition of MWCNTs, which is obvious from the electrochemical performances of GP5C and GP10C samples. The improved electrochemical performances of the composite can be ascribed to the fact that CNTs prevent the restacking of rGO sheets and hence facilitate the electrolytic ions to move deeper into the film samples. As the amount of CNTs is increased beyond the optimum value, specific capacitance decreases, which can be ascribed to the limited dispersibility and poor specific capacitance (~ 20 F/g) of MWCNTs [53, 54].

Moreover, the specific capacitance of all the synthesized films decreases with an increase in the current density because the diffusion of electrolytic ions into the film electrodes becomes slower at higher current density values. Figure 8d shows the Nyquist plots of all the electrodes, indicating that with an increase of MWCNT content, internal resistance starts to decrease. The internal resistance is the Ohmic resistance, which consists of ionic resistance of electrolyte, inherent resistance of substrate and active electrode material, and contact resistance at the active electrode material and substrate interface. GP10C film electrode demonstrates the smallest internal resistance (1.14 Ω), while the internal resistances for rGO, GP5C, and GP15C are found to be about 2.2, 1.41, and 1.19 Ω, respectively. The smaller value of internal resistance for GP10C film can be ascribed to the better contact and its higher electrical conductivity. The “knee” frequency is defined as the highest frequency value at which impedance of the system is dominated by the capacitive nature [55]. It is related to the diffusion coefficient and effective diffusion length of the active electrode material. Further, at the frequencies higher than knee frequency, the electrolytic ions come across semi-infinite diffusion and finite diffusion at the frequencies lower than this [56, 57]. The knee frequency values for GP5C, GP10C, and GP15C are 1.37, 1.49, and 1.10 Hz, respectively. The higher knee frequency value for GP10C implies that lesser time is required by the charge species to accumulation at the interface for this sample. Further, it is well documented that larger semicircle at higher-to-medium frequency region corresponds to the larger charge-transfer resistance (Rct) [31, 58]. The Rct for GP15C film seems to be quite higher than that of GP10C, that may be due to its lower electrical conductivity and higher contact resistance with aqueous electrolyte [59].

**Fig. 9:**
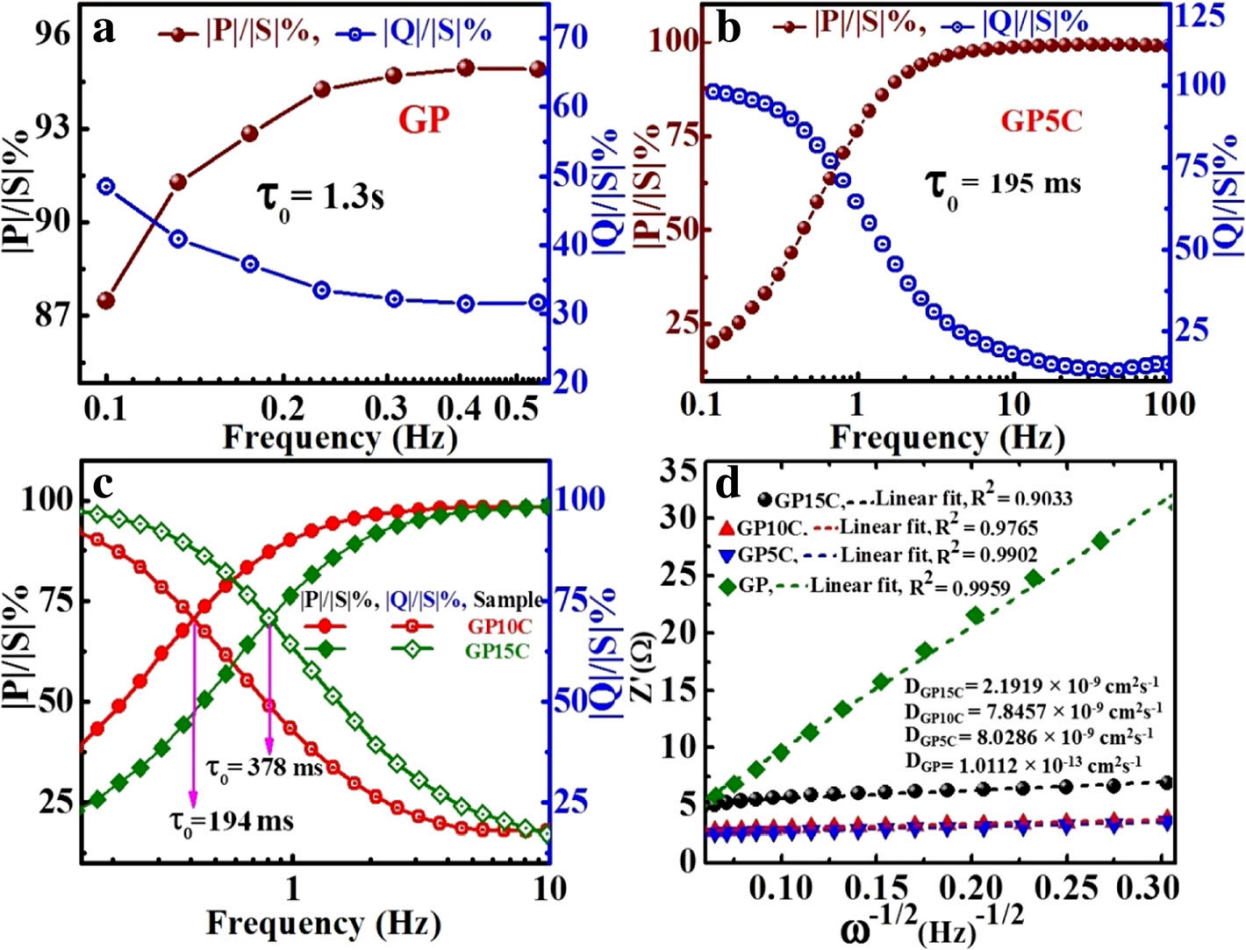
**a**–**c** are the normalized real part |P|/|S| and imaginary part |Q|/|S| of the complex power as a function of frequency for GP, GP5C, and GP10C, respectively, and **d** Randles plots of all the synthesized electrodes

Further, EIS data can be used to find out the relaxation time constant (*τ*_0_) of the devices like supercapacitors in terms of complex power with the help of Eqs. (8) and (9). Relaxation time constant (*τ*_0_) is an important parameter and considered as a factor of merit for a supercapacitor. To determine the relaxation time constant, normalized imaginary factor (|*Q*|/|*S*|) and real factor (|*P*|/|*S*|) of power are plotted vs. frequency (in logarithmic scale) (Fig. 9). Both these two curves cross each other at a point called resonance frequency (*f*_°_), which is utilized to calculate the relaxation time of a supercapacitor using the following formula: *τ*_0_ = 1/2*πf*_0_ [49]. From the graphs, we observe that at a higher frequency, |*P*|/|*S*| attains maximum value, which implies maximum power dissipates in the system, i.e., supercapacitor behaves similar to pure resistor. As the frequency decreases, |*P*|/|*S*| decreases up to a point at which |*Q*|/|*S*| attains the highest value. At this point, supercapacitor works similar to a pure capacitor. Evidently, for all the tested films GP(rGO), GP5C, GP10C, and GP15C, both the |P|/|S| and |Q|/|S| curves act contrarily with frequency variation and cross each other at resonance frequency (*f*_°_). The relaxation time constant values for GP, GP5C, GP10C, and GP15C as calculated using resonance frequencies are 1.3 s, 196 ms, 194 ms, and 378 ms, respectively. After adding MWCNTs in the rGO film, relaxation time decreases remarkably. This may be due to the fact that CNTs prevent the restacking of rGO sheets and hence allow the electrolytic ions to move faster into the film. As the amount of MWCNTs increases further (15 wt%) in the rGO film, increment in the relaxation time constant is observed. This can be ascribed to the smaller diameter of MWCNTs (10–20 nm) that offers higher ionic diffusion resistance, which become significant as the amount of MWCNTs is increased beyond optimum value [60, 61]. EIS results can also be used to determine the diffusion coefficients of the synthesized films for electrolytic ions (Fig. 9d). The calculated diffusion coefficients (*D*_a_) of electrolytic ions at the interfacial region using Eq. (7) come out to be 1.0112 × 10^−13^, 8.0286 × 10^−9^, 7.8457 × 10^−9^, and 2.1919 × 10^−9^ for GP, GP5C, GP10C, and GP15C, respectively, in 2 M KOH. It can be seen that the relaxation time constant and diffusion coefficient of GP5C and GP10C are almost the same, but the Cs and rate capability of GP10C is much better than those of GP5C. The small relaxation time constant and high diffusion coefficient of GP10C film electrode, allow it to deliver stored energy quickly, and high specific capacitance make it desirable for engineering high-power capacitors.

**Fig. 10:**
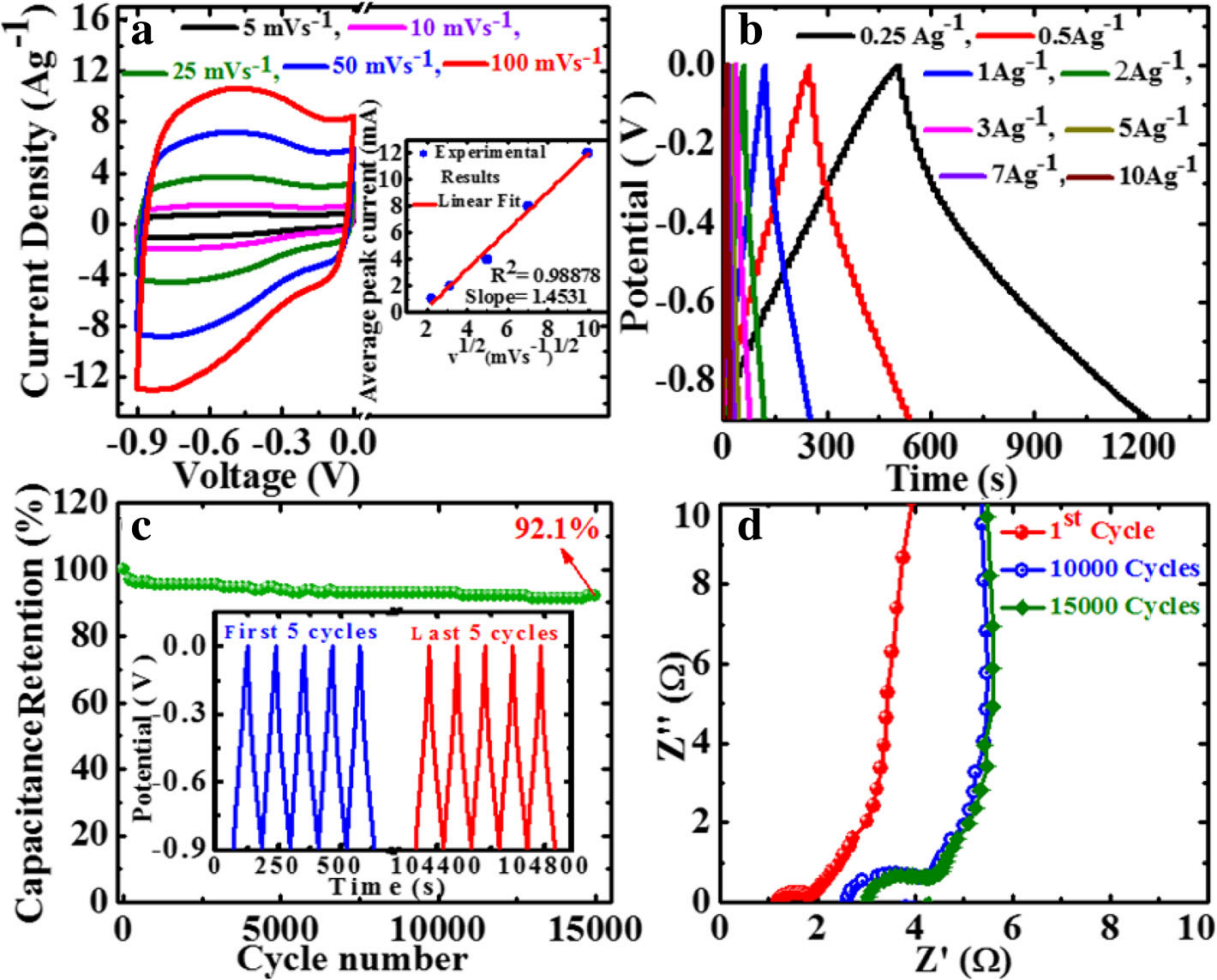
Electrochemical performance of GP10C in 2 M KOH electrolyte **a** CV curves at the scan rate of 5, 10, 25, 50, and 100 mVs^−1^; **b** GCD curves at the current densities of 0.25, 0.5, 1.0, 2.0, 3.0, 5.0, 7.0, and 10 Ag^−1^; **c** cyclic stability performance for GP10C electrode at 2 Ag^−1^ and inset shows the GCD curves of first and last 5 cycles; and **d** Nyquist plot for the GP10C and inset shows the EIS performance during 1st, 10,000 and 15,000 cycles

**Fig. 11:**
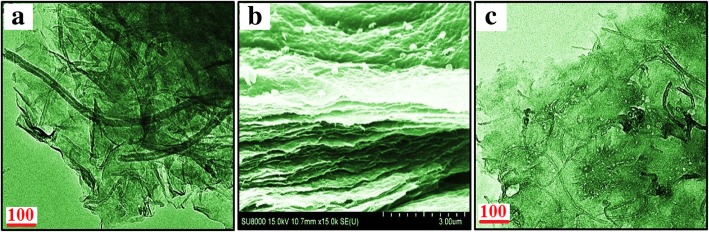
**a** TEM images of the CP10C electrode before long cycle test and **b** FESEM and **c** TEM images of the CP10C after 15,000 cycles

From the above results, GP10C film-based supercapacitor electrode exhibits the best electrochemical properties among the synthesized films. Therefore, we investigate its electrochemical performance in detail. Figure 10a indicates the CV curves of GP10C at 5, 10, 25, 50, and 100 mVs^−1^ in the potential range − 0.9 V to 0.0 V vs Ag/AgCl reference electrode. It is shown that all the CV curves possess almost rectangular and symmetric shape, indicating the perfect EDL capacitive behavior and fast charging/discharging characteristics. The inset in Fig. 10a shows nearly a linear relationship between average peak current and the square root of the scanning rate with correlation coefficient *R*^2^ = 0.98878. This phenomenon indicates that the electrochemical process in the film is a diffusion-controlled process [62]. Figure 10b represents the GCD curves of GP10C evaluated at 0.25 to 10 Ag^−1^ in − 0.9 to 0.0 V. During the charge/discharge process, the corresponding curves also verify that the charging curve of GP10C is almost symmetric to its corresponding discharging curve. To evaluate the durability of the GP10C, the long cycle test was carried out in 2 M KOH electrolyte at 2 Ag^−1^. Figure 10c depicts the long cycle stability, which is another important parameter to examine the electrochemical performance of an electrode material. After 15,000 cycles, GP10C electrode exhibits excellent retention of 92.5%. The inset in Fig. 10c shows first and last 5 successive cycles. It demonstrates that even after 15,000 cycles, the electrode maintains good symmetric charge/discharge characteristic features, which verify its excellent electrochemical durability. Figure 10d represents the Nyquist plots of the GP10C electrode recorded during long cycle test. It can be observed that the value of internal resistance goes higher during cycling process from the first cycle to 15,000 cycles. GP10C electrode shows lowest internal resistance (1.12 Ω) during the first cycle and after 10,000 and 15,000 cycles, as the electrochemical active sites in the electrode are slowly consumed, the values of internal resistance increases from 2.64 to 3.04 Ω, respectively. As a consequence of it, CV value decreases slowly and repeatedly during electrochemical cycling (Fig. 10c). Furthermore, to find out any morphological changes in the GP10C film electrode after long cycle test, we performed ex situ studies (FESEM and TEM), and the results are shown in Fig. 11. Figure 11a shows the TEM images of GP10C electrode before the long cycle test, while Figs. 11b and c represent the FESEM and TEM images of the GP10C after 15,000 cycles. We can see that the morphology of the GP10C electrode does not change even after 15,000 cycles, which reveals the sustained chemical stability of the film. The observed capacitance of GP10C film electrode is higher than those of several recently reported free-standing graphene-based supercapacitor electrodes as shown in Table 1.

**Table 1:** Electrochemical performance of the GP10C electrode compared with other recently reported results in the literature

Material	Method	Specific capacitance	Scan rate/current density	Electrolyte	Ref.
rGO/PANI/rGO paper	Vacuum filtration	55 Fg^−1^	1 Ag^−1^	1 M H_2_SO_4_	[20]
rGO paper	Vacuum filtration	80 Fg^−1^	0.5 Ag^−1^	6 M KOH	[21]
rGO paper	Vacuum filtration	130 Fg^−1^	0.1 Ag^−1^	6 M KOH	[22]
rGO/Fe_2_O_3_ paper	Vacuum filtration	178 Fcm^−1^	1 mVs^−1^	3 M KOH	[23]
Graphene/MnO_2_ paper	Vacuum filtration	217 Fg^−1^	0.1 Ag^−1^	1 M Na_2_SO_4_	[26]
P-rGO paper	Self- assembled on Cu foil	100 Fg^−1^	100 mVs^−1^	1 M H_2_SO_4_	[63]
MnO_2_ NW/CNT paper	Vacuum filtration	167.5 Fg^−1^	0.077 Ag^−1^	0.1 M Na_2_SO_4_	[64]
Graphene/CNT paper	Vacuum filtration	126 Fg^−1^	1 Ag^−1^	6 M KOH	[65]
rGO/CB paper	Vacuum filtration	95.5 Fg^−1^	5 mVs^−1^	1 M H_2_SO_4_	[66]
GP10C film	Self-assembled plus reduction	200 Fg^−1^	0.25 Ag^−1^	2 M KOH	In this work

### Electrochemical Performance of Symmetrical Supercapacitor

**Fig. 12:**
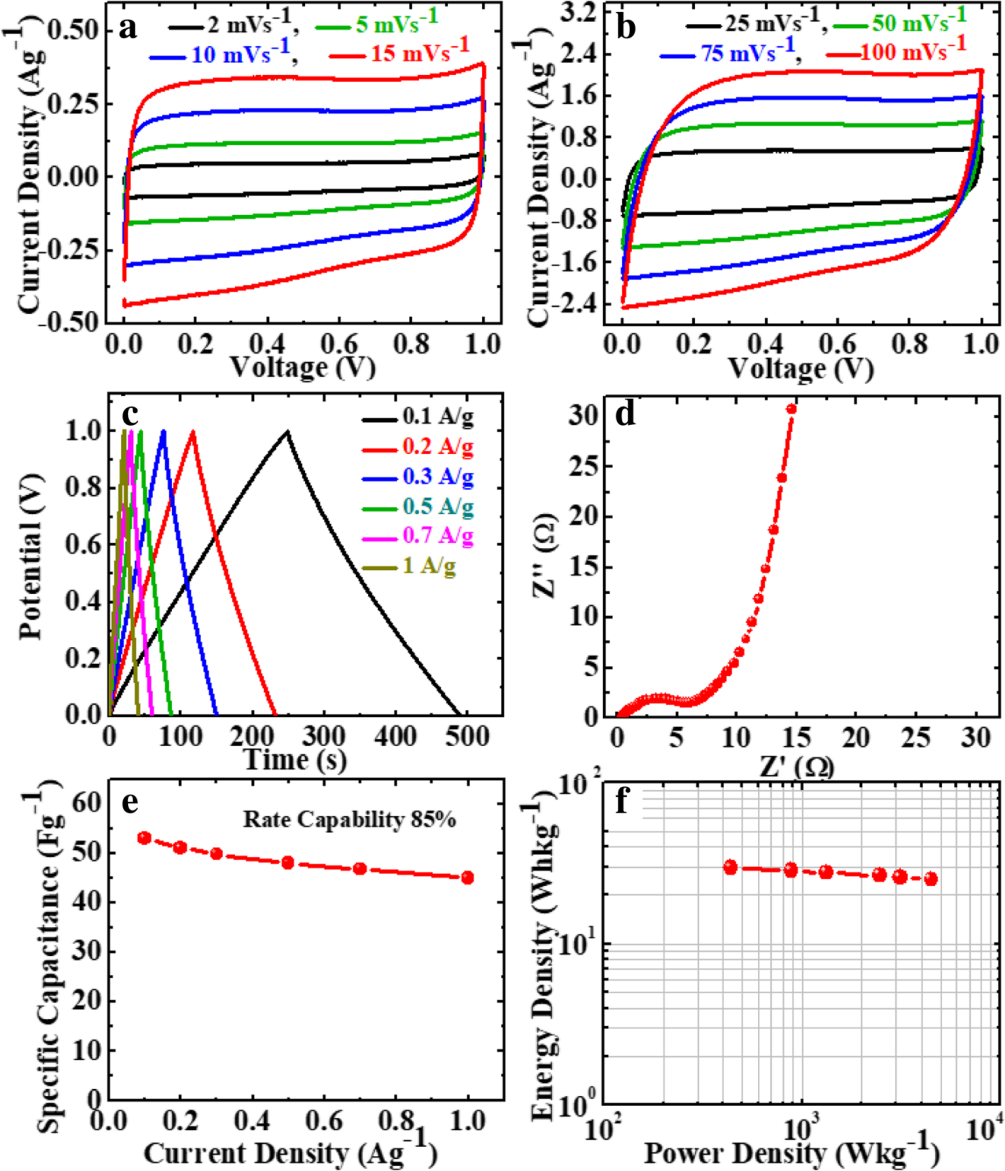
Electrochemical performance of GP10C/KOH/GP10C symmetrical coin supercapacitor cell **a**, **b** CV curves of GP10C/KOH/GP10C coin cell at 2, 5, 10, 15, 25, 50, 75, and 100 mVs^−1^, **c** Nyquist plot, **d** GCD curves of the device at different current densities, **e** SC at different current densities, **f** Ragone plot

Further, to investigate the practical application of the GP10C film, we made a symmetric coin cell supercapacitor using two GP10C electrodes of identical weight separated by a separator in 2 M KOH aqueous electrolyte. Figures 12a and b show the CV profiles of the device at the scan rates of 2, 5, 10, 15, 25, 50, 75, and 100 mVs^−1^. We can observe nearly identical rectangular shape, which implies the perfect EDLC behavior of the supercapacitor. Figure 12c represents the linear GCD curves at all current densities demonstrating the high rate response of the device. Moreover, the smaller internal resistance (0.4 Ω) of the coin cell indicates better charge transportation in the supercapacitor (Fig. 12d). The calculated specific capacitances from CVs of the device (Fig. 12e) are 53, 51, 49.8, 48, 46.7, and 45 Fg^−1^ at 0.1, 0.2, 0.3, 0.5, 0.7, and 1.0 Ag^−1^, respectively. From the capacitance profile (Fig. 12e), it is clearly shown that the device retains 85% of its initial capacitance value at current density 0.1 Ag^−1^ up to 1 Ag^−1^, i.e., good rate capability. Additionally, we calculate the energy density (Whkg^−1^) and power density (Wkg^−1^) of the device using equations given below [8, 9]:

8 $$ E=\frac{\mathrm{Cs}}{2\times 3.6}{\left(\Delta  V\right)}^2 $$

9 $$ P=\frac{E}{\Delta  t}\times 3600 $$

where *Cs* is the SC calculated from the GDC curves, *∆V* is the potential window, *t* is the discharge time (s).

The device exhibits maximum and minimum energy densities of 29.4 and 25.0 Whkg^−1^ at power densities of 439 and 4500 Wkg^−1^, respectively (Fig. 12f).

**Fig. 13:**
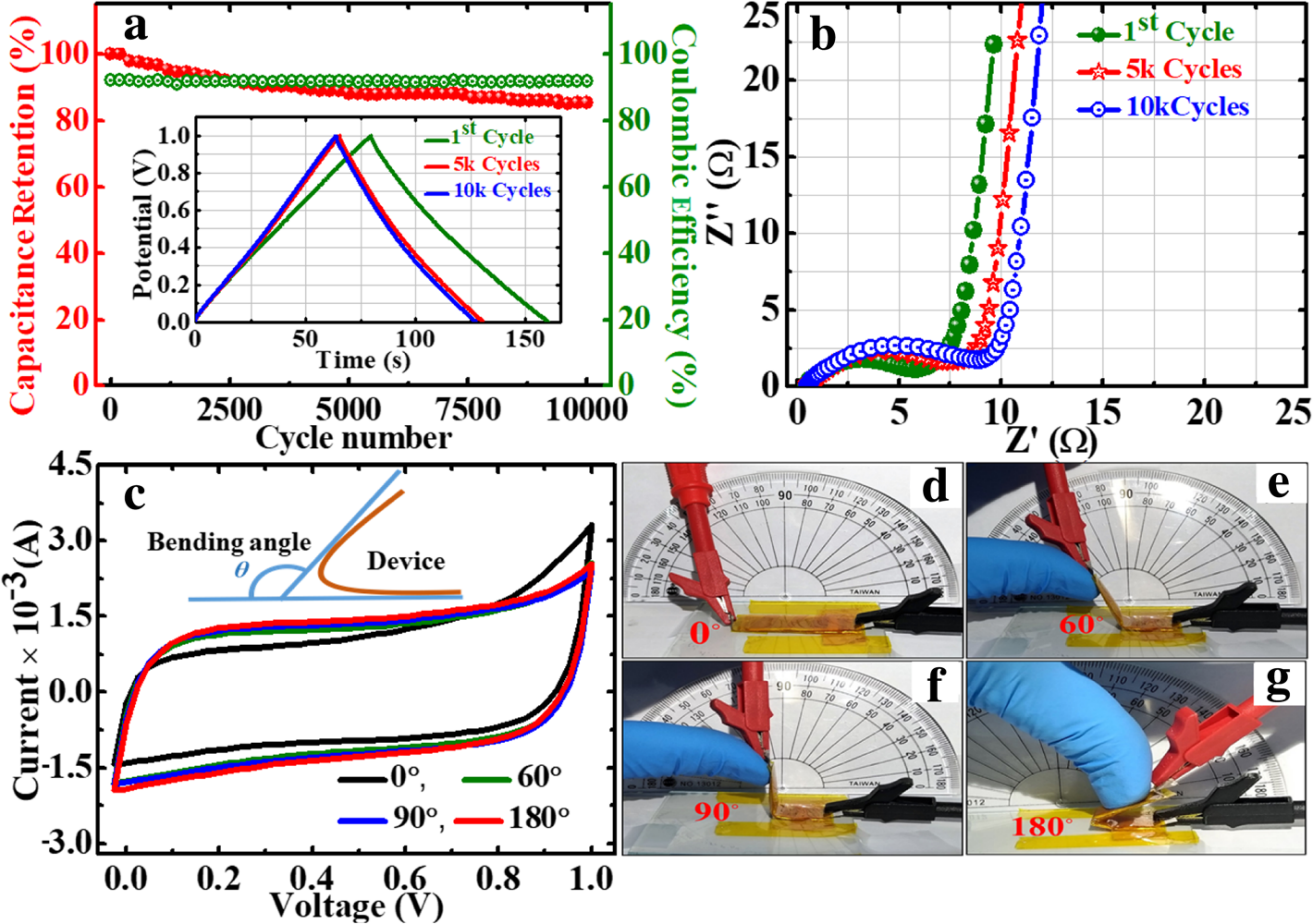
The long cycle performance of GP10C/KOH/GP10C symmetrical coin cell. **a** Cyclic stability and columbic efficiency recorded at 0.3 Ag^−1^ for 10,000 successive cycles, and inset shows the GCD profiles of 1st, 5000th and 10,000th GCD cycles. **b** Nyquist plots recorded just after 1st, 5000th and 10,000th cycles, and inset shows a red LED light up by single coin cell. **c** The CV curves at a scan rate of 20 mVs^−1^ of symmetrical solid state flexible device using gel polymer electrolyte under different bending angles. Digital photographs of the device under different bending angles, **d** 0°, **e** 60°, **f** 90°,and **g** 180°, respectively

This symmetric device shows excellent retention of ~ 85% and columbic efficiency of 92% after 10,000 successive cycles at 0.3 Ag^−1^ (Fig. 13a). The excellent cyclability of the device can be ascribed to the electrochemical stability of the active electrode material. In the GP10C nanocomposite film, the optimum amount of MWCNTs mainly prevents the restacking of rGO sheets and thus offers a more exposed area to the electrolytic ions for surface adsorption. This also strengthens the material structure to resist the structural deformation upon cycling. The ex situ TEM and FESEM micrographs of the tested electrode after 15,000 cycles (Fig. 11a–c) verify the behavior that the morphology of GP10C electrode remains the same even after 15,000 cycles, which reveals the sustained chemical stability of the synthesized composite film. The inset in Fig. 13a shows the GCD profiles of 1st, 5000th, and 10,000th charge-discharge cycles, indicating the symmetric charge/discharge characteristic features of the device. The high retention at even after 10,000 continuous long cycles verifies its outstanding electrochemical durability. Figure 13b depicts the Nyquist plots of the device during long cycle test, implies that with repeated cycles, the Warburg region in the middle frequency region is increasing. It can be attributed to the consumption of active sites presented in the active material of the supercapacitor electrodes during a long cyclic test, which results in an increase of the internal resistance of the device. The inset (Fig. 13b) shows that our symmetric coin cell can light up a red LED**.** Further, our designed FSSSD using GP10C flexible film electrodes and gel electrolyte depicts no significant changes in the shape of CV curves when bending the device at angles from 0 to 180° at a scan rate of 20 mVs^−1^ (Fig. 13c). Digital photographs of the device under the bending angles 0°, 60°, 90°, and 180° are shown in Fig. 13d–g, respectively.

The above results prove the potential applications of our synthesized GP10C film for the supercapacitors. Moreover, this facile approach may open future prospects for energy storage devices application.

## Conclusions

In summary, simple and cost-effective rGO/MWCNT flexible film electrodes were synthesized via simplest chemical route. The effects of MWCNT addition on the electrochemical performance of rGO/MWCNT nanocomposite films were investigated in different alkaline electrolytes, KOH, LiOH, and NaOH. Based on experimental findings, GP10C exhibits the best electrochemical performance in 2 M KOH with SC of 200 Fg^−1^. This synthesized film electrode demonstrates excellent durability with 92% retention after 15,000 long cycle test, small relaxation time constant (~ 194 ms), and high diffusion coefficient (7.8457 × 10^−9^ cm^2^ s^−1^) in 2 M KOH aqueous electrolyte. The superior electrochemical performance of GP10C can be attributed to the smaller hydration sphere radius and higher ionic conductivity of K^+^ cations. The symmetric coin supercapacitor cell using GP10C as both anode and cathode and 2 M KOH as electrolyte exhibits perfect EDLC behavior with maximum energy and power densities of 29.4 Whkg^−1^ and 4500 Wkg^−1^, respectively. Our symmetric cell demonstrates excellent retention of 85.3%, and columbic efficiency of 92% after 10,000 successive cycles at 0.3 Ag^−1^. Further, the designed FSSSD using GP10C flexible film electrodes and gel electrolyte depicts no significant changes in the shape of CV curves when bending the device at angles from 0 to 180° at 20 mVs^−1^. We believe that our rGO/MWCNT nanocomposite film is suitable for practical applications and appropriate for designing high capacitive energy storage (supercapacitors or Li-batteries), conversion, and wearable devices.

## Data Availability

All data and materials are fully available without resection.

## Abbreviations

BET: Brunauer-Emmett-Teller
CV: Cyclic voltammetry
EDLC: Electrical double-layer capacitor
EIS: Electrochemical impedance spectroscopy
FESEM: Field-emission electron microscope
GCD: Galvanostatic charge/discharge
GO: Graphene oxide
GP: rGO/MWCNT film with 0% CNT ratio
GP10C: rGO/MWCNT film with 10% CNT ratio
GP15C: rGO/MWCNT film with 15% CNT ratio
GP5C: rGO/MWCNT film with 5% CNT ratio
HI: Hydriodic acid solution
MWCNTs: Multiwall carbon nanotubes
PVA: Polyvinyl alcohol
rGO: Reduced graphene oxide
SC: Specific capacitance
TEM: Transmission electron microscope
TGA: Thermogravimetric analyzer
XRD: X-ray diffraction
